# Genetically elevated high-density lipoprotein cholesterol through the cholesteryl ester transfer protein gene does not associate with risk of Alzheimer's disease

**DOI:** 10.1016/j.dadm.2018.08.008

**Published:** 2018-09-22

**Authors:** Gina M. Peloso, Sven J. van der Lee, International Genomics of Alzheimer's Project (IGAP), R. Sims, S.J. van der Lee, A.C. Naj, C. Bellenguez, N. Badarinarayan, J. Jakobsdottir, B.W. Kunkle, A. Boland, R. Raybould, J.C. Bis, E.R. Martin, B. Grenier-Boley, S. Heilmann-Heimbach, V. Chouraki, A.B. Kuzma, K. Sleegers, M. Vronskaya, A. Ruiz, R.R. Graham, R. Olaso, P. Hoffmann, M.L. Grove, B.N. Vardarajan, M. Hiltunen, M.M. Nöthen, C.C. White, K.L. Hamilton-Nelson, J. Epelbaum, W. Maier, S.H. Choi, G.W. Beecham, C. Dulary, S. Herms, A.V. Smith, C.C. Funk, Derbois, A.J. Forstner, S. Ahmad, H. Li, D. Bacq, D. Harold, C.L. Satizabal, O. Valladares, A. Squassina, R. Thomas, J.A. Brody, L. Qu, P. Sánchez-Juan, T. Morgan, F.J. Wolters, Y. Zhao, F.S. Garcia, N. Denning, M. Fornage, J. Malamon, M.C.D. Naranjo, E. Majounie, T.H. Mosley, B. Dombroski, D. Wallon, M.K. Lupton, J. Dupuis, P. Whitehead, L. Fratiglioni, C. Medway, X. Jian, S. Mukherjee, L. Keller, K. Brown, H. Lin, L.B. Cantwell, F. Panza, B. McGuinness, S. Moreno-Grau, J.D. Burgess, V. Solfrizzi, P. Proitsi, H.H. Adams, M. Allen, D. Seripa, P. Pastor, L.A. Cupples, N.D. Price, D. Hannequin, A. Frank-García, D. Levy, P. Chakrabarty, P. Caffarra, I. Giegling, A.S. Beiser, V. Giedraitis, H. Hampel, M.E. Garcia, X. Wang, L. Lannfelt, P. Mecocci, G. Eiriksdottir, P.K. Crane, F. Pasquier, V. Boccardi, I. Henández, R.C. Barber, M. Scherer, L. Tarraga, P.M. Adams, M. Leber, Y. Chen, M.S. Albert, S. Riedel-Heller, V. Emilsson, D. Beekly, A. Braae, R. Schmidt, D. Blacker, C. Masullo, H. Schmidt, R.S. Doody, G. Spalletta, W.T. Longstreth, T.J. Fairchild, P. Bossù, O.L. Lopez, M.P. Frosch, E. Sacchinelli, B. Ghetti, Q. Yang, R.M. Huebinger, F. Jessen, S. Li, M.I. Kamboh, J. Morris, O. Sotolongo-Grau, M.J. Katz, C. Corcoran, M. Dunstan, A. Braddel, C. Thomas, A. Meggy, R. Marshall, A. Gerrish, J. Chapman, M. Aguilar, S. Taylor, M. Hill, M.D. Fairén, A. Hodges, B. Vellas, H. Soininen, I. Kloszewska, M. Daniilidou, J. Uphill, Y. Patel, J.T. Hughes, J. Lord, J. Turton, A.M. Hartmann, R. Cecchetti, C. Fenoglio, M. Serpente, M. Arcaro, C. Caltagirone, M.D. Orfei, A. Ciaramella, S. Pichler, M. Mayhaus, W. Gu, A. Lleó, J. Fortea, R. Blesa, I.S. Barber, K. Brookes, C. Cupidi, R.G. Maletta, D. Carrell, S. Sorbi, S. Moebus, M. Urbano, A. Pilotto, J. Kornhuber, P. Bosco, S. Todd, D. Craig, J. Johnston, M. Gill, B. Lawlor, A. Lynch, N.C. Fox, J. Hardy, R.L. Albin, L.G. Apostolova, S.E. Arnold, S. Asthana, C.S. Atwood, C.T. Baldwin, L.L. Barnes, S. Barral, T.G. Beach, J.T. Becker, E.H. Bigio, T.D. Bird, B.F. Boeve, J.D. Bowen, A. Boxer, J.R. Burke, J.M. Burns, J.D. Buxbaum, N.J. Cairns, C. Cao, C.S. Carlson, C.M. Carlsson, R.M. Carney, M.M. Carrasquillo, S.L. Carroll, C.C. Diaz, H.C. Chui, D.G. Clark, D.H. Cribbs, E.A. Crocco, C. DeCarli, M. Dick, R. Duara, D.A. Evans, K.M. Faber, K.B. Fallon, D.W. Fardo, M.R. Farlow, S. Ferris, T.M. Foroud, D.R. Galasko, M. Gearing, D.H. Geschwind, J.R. Gilbert, N.R. Graff-Radford, R.C. Green, J.H. Growdon, R.L. Hamilton, L.E. Harrell, L.S. Honig, M.J. Huentelman, C.M. Hulette, B.T. Hyman, G.P. Jarvik, E. Abner, L.W. Jin, G. Jun, A. Karydas, J.A. Kaye, R. Kim, N.W. Kowall, J.H. Kramer, F.M. LaFerla, J.J. Lah, J.B. Leverenz, A.I. Levey, G. Li, A.P. Lieberman, K.L. Lunetta, C.G. Lyketsos, D.C. Marson, F. Martiniuk, D.C. Mash, E. Masliah, W.C. McCormick, S.M. McCurry, A.N. McDavid, A.C. McKee, M. Mesulam, B.L. Miller, C.A. Miller, J.W. Miller, J.C. Morris, J.R. Murrell, A.J. Myers, S. O'Bryant, J.M. Olichney, V.S. Pankratz, J.E. Parisi, H.L. Paulson, W. Perry, E. Peskind, A. Pierce, W.W. Poon, H. Potter, J.F. Quinn, A. Raj, M. Raskind, B. Reisberg, C. Reitz, J.M. Ringman, E.D. Roberson, E. Rogaeva, H.J. Rosen, R.N. Rosenberg, M.A. Sager, A.J. Saykin, J.A. Schneider, L.S. Schneider, W.W. Seeley, A.G. Smith, J.A. Sonnen, S. Spina, R.A. Stern, R.H. Swerdlow, R.E. Tanzi, T.A. Thornton-Wells, J.Q. Trojanowski, J.C. Troncoso, V.M. Van Deerlin, L.J. Van Eldik, H.V. Vinters, J.P. Vonsattel, S. Weintraub, K.A. Welsh-Bohmer, K.C. Wilhelmsen, J. Williamson, T.S. Wingo, R.L. Woltjer, C.B. Wright, C.E. Yu, L. Yu, F. Garzia, F. Golamaully, G. Septier, S. Engelborghs, R. Vandenberghe, P.P. De Deyn, C.M. Fernadez, Y.A. Benito, H. Thonberg, C. Forsell, L. Lilius, A. Kinhult-Stählbom, L. Kilander, R. Brundin, L. Concari, S. Helisalmi, A.M. Koivisto, A. Haapasalo, V. Dermecourt, N. Fievet, O. Hanon, C. Dufouil, A. Brice, K. Ritchie, B. Dubois, J.J. Himali, C.D. Keene, J. Tschanz, A.L. Fitzpatrick, W.A. Kukull, M. Norton, T. Aspelund, E.B. Larson, R. Munger, J.I. Rotter, R.B. Lipton, M.J. Bullido, A. Hofman, T.J. Montine, E. Coto, E. Boerwinkle, R.C. Petersen, V. Alvarez, F. Rivadeneira, E.M. Reiman, M. Gallo, C.J. O'Donnell, J.S. Reisch, A.C. Bruni, D.R. Royall, M. Dichgans, M. Sano, D. Galimberti, P. St George-Hyslop, E. Scarpini, D.W. Tsuang, M. Mancuso, U. Bonuccelli, A.R. Winslow, A. Daniele, C.K. Wu, O. Peters, B. Nacmias, M. Riemenschneider, R. Heun, C. Brayne, D.C. Rubinsztein, J. Bras, R. Guerreiro, A. Al-Chalabi, C.E. Shaw, J. Collinge, D. Mann, M. Tsolaki, J. Clarimón, R. Sussams, S. Lovestone, M.C. O'Donovan, M.J. Owen, T.W. Behrens, S. Mead, A.M. Goate, A.G. Uitterlinden, C. Holmes, C. Cruchaga, M. Ingelsson, D.A. Bennett, J. Powell, T.E. Golde, C. Graff, P.L. De Jager, K. Morgan, N. Ertekin-Taner, O. Combarros, B.M. Psaty, P. Passmore, S.G. Younkin, C. Berr, V. Gudnason, D. Rujescu, D.W. Dickson, J.F. Dartigues, A.L. DeStefano, S. Ortega-Cubero, H. Hakonarson, D. Campion, M. Boada, J.K. Kauwe, L.A. Farrer, C. Van Broeckhoven, M.A. Ikram, L. Jones, J.L. Haines, C. Tzourio, L.J. Launer, V. Escott-Price, R. Mayeux, J.F. Deleuze, N. Amin, P.A. Holmans, M.A. Pericak-Vance, P. Amouyel, C.M. van Duijn, A. Ramirez, L.S. Wang, J.C. Lambert, S. Seshadri, J. Williams, G.D. Schellenberg, Anita L. Destefano, Sudha Seshardi

**Affiliations:** a Department of Biostatistics, Boston University School of Public Health, Boston, MA, USA; b Department of Epidemiology, Erasmus MC University Medical Center, Rotterdam, Netherlands; c NHLBI's Framingham Heart Study, Framingham, MA, USA; d Department of Neurology, Boston University School of Medicine, Boston, MA, USA; e Glenn Biggs Institute for Alzheimer's and Neurodegenerative Diseases, University of Texas Health Sciences Center, San Antonio, TX, USA

**Keywords:** Genetics, HDL-C, Single nucleotide polymorphisms, Instrumental variables, Cholesteryl ester transfer protein

## Abstract

**Introduction:**

There is conflicting evidence whether high-density lipoprotein cholesterol (HDL-C) is a risk factor for Alzheimer's disease (AD) and dementia. Genetic variation in the cholesteryl ester transfer protein (*CETP*) locus is associated with altered HDL-C. We aimed to assess AD risk by genetically predicted HDL-C.

**Methods:**

Ten single nucleotide polymorphisms within the *CETP* locus predicting HDL-C were applied to the International Genomics of Alzheimer's Project (IGAP) exome chip stage 1 results in up 16,097 late onset AD cases and 18,077 cognitively normal elderly controls. We performed instrumental variables analysis using inverse variance weighting, weighted median, and MR-Egger.

**Results:**

Based on 10 single nucleotide polymorphisms distinctly predicting HDL-C in the *CETP* locus, we found that HDL-C was not associated with risk of AD (*P* > .7).

**Discussion:**

Our study does not support the role of HDL-C on risk of AD through HDL-C altered by *CETP*. This study does not rule out other mechanisms by which HDL-C affects risk of AD.

## Highlights

• *CETP* SNPs were not associated with AD in a large sample of AD cases/controls.
• Genetically predicted HDL-C through *CETP* does not associate with AD.

## 1 Introduction

Alzheimer's disease (AD) is an incurable neurological disease affecting more than 5 million individuals living in the United States [1]. A potential connection between cholesterol levels and risk of AD and dementia has been suggested [2]. This is important as cholesterol levels in blood can be modified. However, there is conflicting epidemiological evidence whether high-density lipoprotein cholesterol (HDL-C) levels, which is considered the beneficial cholesterol, is a risk factor for AD and dementia. In a prospective study of approximately 7000 French individuals, there was no association of HDL-C with incident all-cause dementia or AD [3]. On the other hand, in the same French cohort, an association was observed in men between incident all-cause dementia, but not AD [4], and in a study of 75,000 individuals in Denmark, HDL-C was associated with both all-cause dementia and AD [5]. When evidence was combined from multiple published studies, late-life HDL-C was not associated with all-cause dementia or AD [6].

Cholesteryl ester transfer protein (CETP) is involved in the exchange of cholesteryl esters and phospholipids between HDLs and other lipoproteins. Increased plasma HDL-C and plasma CETP is linked to a reduced risk of cardiovascular disease [7], [8]. Common single nucleotide polymorphisms (SNPs) in the *CETP* locus have been strongly linked to altered plasma lipid levels [9]. Furthermore, protein-truncating variants (i.e., nonsense, frameshift, and splice site variants) in *CETP* are associated with higher HDL-C, lower low-density lipoprotein cholesterol, lower triglycerides, and lower risk of coronary heart disease [10]. The Global Lipids Genetic Consortium has reported 10 variants on the exome array that are significantly and distinctly associated with HDL-C [11]. These SNPs in *CETP* with large effects on HDL-C can be used as tools to elucidate whether there is a causal role of HDL-C on AD. In this study, we use instrumental variable analysis with genetic instruments (i.e., Mendelian Randomization) to predict whether high HDL-C through *CETP* is associated with AD by answering the questions: (1) Do *CETP* SNPs associate with AD risk?; and (2) Does genetically predicted high HDL-C through *CETP* associate with risk of AD?

## 2 Methods

### 2.1 Association statistics

We obtained results for 10 SNPs previously shown to be strongly and distinctly associated with HDL-C in the *CETP* region (Table 1) from the Global Lipids Genetic Consortium exome chip results in up to 316,391 individuals, mostly of European origin [10], [11]. HDL-C was measured by standard protocols, and the majority of individuals were fasting [11]. The analysis of HDL-C was adjusted for age, sex, population stratification, and relatedness, where appropriate, and inverse normalized residuals were used as outcomes. Results were meta-analyzed across cohorts using an additive model. The *CETP* region was defined as being within 1 MB of an indexed *CETP* SNP (rs3764261).

**Table 1:** GLGC and IGAP exome chip results for 10 SNPs in the *CETP* region distinctly associated with plasma HDL-C levels

SNP	Protein	Effect allele	Frequency	HDL-C			AD model 1			AD model 2		
				β	SE	*P* value	β	SE	*P* value	β	SE	*P* value
rs2303790	ASP459GLY	G	0.08%	0.366	0.047	5 × 10^−15^	0.280	1.419	0.844	−0.801	1.703	0.638
rs34065661	ALA15GLY	G	0.48%	0.435	0.020	6 × 10^−103^	0.034	0.307	0.912	0.142	0.334	0.670
rs247616	Intergenic	T	30.83%	0.242	0.003	<1 × 10^−323^	−0.004	0.018	0.812	0.003	0.020	0.879
rs3764261	Intergenic	A	31.27%	0.239	0.003	<1 × 10^−323^	0.004	0.018	0.832	−0.003	0.020	0.880
rs173539	Intergenic	T	32.26%	0.230	0.003	<1 × 10^−323^	−0.008	0.018	0.679	−0.001	0.020	0.959
rs5882	VAL422ILE	G	35.04%	0.092	0.003	6 × 10^−241^	0.002	0.018	0.908	−0.009	0.020	0.640
rs9989419	Intergenic	G	60.09%	0.131	0.003	<1 × 10^−323^	−0.002	0.018	0.892	0.002	0.020	0.906
rs9939224	Intronic	G	78.73%	0.205	0.003	<1 × 10^−323^	0.026	0.021	0.220	0.029	0.023	0.201
rs7499892	Intronic	C	80.83%	0.230	0.003	<1 × 10^−323^	−0.030	0.022	0.187	−0.035	0.024	0.151
rs5880	ALA390PRO	G	95.19%	0.258	0.007	4 × 10^−321^	−0.017	0.045	0.705	0.002	0.050	0.970

Abbreviations: AD, Alzheimer's deisease; GLGC, Global Lipids Genetic Consortium; IGAP, International Genomics of Alzheimer's Project; PC, principal components; SNPs, single nucleotide polymorphisms; CETP, cholesteryl ester transfer protein; HDL-C, high-density lipoprotein cholesterol.

NOTE. AD model 1 adjusts for PCs of ancestry only.

NOTE. AD model 2 adjusts for PCs of ancestry, age, and sex.

We obtained results for the 10 identified *CETP* SNPs with AD within the IGAP exome chip results in up to 34,174 individuals from the stage 1 results (up to 16,097 late onset AD cases and 18,077 cognitively normal elderly controls) [12]. Results from multiple consortia were meta-analyzed using an additive model. Two sets of covariates were used in the model of association: principal components (PCs) of ancestry only adjustment (model 1) and PCs, age and sex adjustment (model 2).

Both sets of results were based on exome chip genotypes and aligned to the forward strand.

### 2.2 Statistical analyses

We obtained *CETP*-predicted estimates of the effect of HDL-C on risk of AD from summary statistics using fixed effects inverse variance weighted meta-analysis [13], weighted median method [14], and MR-Egger [15]. A non-zero MR-Egger intercept indicates that the inverse variance weighted estimate may be invalid. We report the odds ratio and 95% confidence intervals per standard deviation of HDL-C.

Our primary analysis is based on the 10 previously reported HDL-C SNPs in the *CETP* locus with the model 1 adjusted AD results. We perform sensitivity analyses on the following sets of SNPs to determine whether the set of SNPs or AD model adjustment influenced the results: (1). 10 HDL-C SNPs with model 2 AD results, (2). 9 HDL-C SNPs with consistent AD effects using model 2 AD results, (3). 7 common HDL-C SNPs with model 2 AD results, (4). 4 nonsynonymous SNPs with model 1 AD results, and (5). 4 nonsynonymous SNPs with model 2 AD results.

All statistical analyses were conducted using the R package Mendelian Randomization in R, version 3.3.1 (R Foundation for Statistical Computing, Vienna, Austria). Only summary statistics were used in this study.

### 2.3 Power calculation

The 10 *CETP* SNPs explain ∼3.5% of variance of HDL-C [11]. We calculated the power to detect an effect of HDL-C through *CETP* on AD through Mendelian randomization [16] given our sample (34,174 individuals, 47% cases) assuming the SNPs explain 3.5% of the variance in HDL-C at an alpha of 0.05 using http://cnsgenomics.com/shiny/mRnd/.

## 3 Results

Ten SNPs in *CETP* region were shown to distinctly and significantly associate with HDL in the Global Lipids Genetic Consortium exome chip analyses (Table 1). Eight of the then *CETP* SNPs have at least a 1/5^th^ standard deviation effect on HDL-C. We associated these 10 SNPs with AD in the IGAP exome chip results. None of the ten *CETP* SNPs were nominally associated with AD (*P* > .05). All the AD results had heterogeneity *P* value > 0.1.

We compared the AD effect estimates between the PC only adjusted model (model 1) and the PC plus age and sex adjusted model (model 2) for the 10 *CETP* region SNPs. We found that one SNP (rs2303790) with discordant effect size between the two AD models (Supplementary Fig. 1A). After removing rs2303790, we found a 0.81 correlation between the effect estimates from the two AD model adjustments (*P* = .0075) (Supplementary Fig. 1B). In both adjustment models, we found a positive trend between each SNPs effect on HDL with its corresponding effect on AD (Supplementary Fig. 1C and D).

Across six sets of results and three statistical models, we found no evidence that genetically increasing HDL-C through *CETP* will lead to an increase in risk of AD (Table 2). MR-Egger intercepts were not found to be different from zero, suggesting that directional pleiotropy was not apparent, and the I^2^ estimate was >99% in all analyses, indicating variability in effects.

**Table 2:** Estimates of the effect of genetically predicted HDL-C through *CETP* on AD

Model	OR (95% CI)	*P* value	MR-Egger intercept (*P* value)	I^2^ (*P* value)
10 HDL SNPs, model 1 AD results				
Inverse variance weighted	0.988 (0.923, 1.059)	0.738		
Weighted Median	0.982 (0.9, 1.07)	0.68		
MR-Egger	0.956 (0.751, 1.217)	0.712	0.007 (0.775)	100% (0.896)
10 HDL SNPs, model 2 AD results				
Inverse variance weighted	0.995 (0.924, 1.071)	0.895		
Weighted Median	0.999 (0.908, 1.099)	0.984		
MR-Egger	1.013 (0.78, 1.318)	0.92	−0.004 (0.885)	100% (0.824)
9 HDL SNPs with consistent AD effects, model 2 AD results				
Inverse variance weighted	0.995 (0.924, 1.073)	0.898		
Weighted Median	0.999 (0.909, 1.097)	0.984		
MR-Egger	1.014 (0.78, 1.318)	0.915	−0.004 (0.881)	99.9% (0.765)
7 Common HDL SNPs, model 2 AD results				
Inverse variance weighted	0.994 (0.921, 1.073)	0.873		
Weighted Median	0.997 (0.906, 1.097)	0.946		
MR-Egger	1.008 (0.771, 1.317)	0.956	−0.003 (0.916)	99.9% (0.557)
4 nonsynonymous SNPs, model 1 AD results				
Inverse variance weighted	0.977 (0.76, 1.257)	0.858		
Weighted Median	0.976 (0.746, 1.276)	0.86		
MR-Egger	0.91 (0.526, 1.576)	0.738	0.010 (0.777)	99.9% (0.953)
4 nonsynonymous SNPs, model 2 AD results				
Inverse variance weighted	0.97 (0.736, 1.279)	0.829		
Weighted Median	0.964 (0.72, 1.289)	0.803		
MR-Egger	1.101 (0.602, 2.014)	0.756	−0.019 (0.645)	99.9% (0.833)

Abbreviations: HDL-C, high-density lipoprotein cholesterol; CETP, cholesteryl ester transfer protein; AD, Alzheimer's deisease; SNPs, single nucleotide polymorphisms; CI, confidence interval.

Given our sample size (34,174 individuals, 47% cases) and the proportion of variance explained in HDL by the *CETP* SNPs (3.5%), we had >80% power to detect an odds ratio > 1.18 or < 0.85 for genetically increased HDL on risk of AD at an alpha of 0.05.

## 4 Discussion

We found that SNPs in the *CETP* locus with a large effect on HDL-C were not associated with risk of AD and that genetically predicted HDL-C, through polymorphisms in the *CETP* locus, does not associate with risk of AD. Our study lends evidence that life-long altered HDL-C through *CETP* is not a causal predictor of risk of AD. Previously, Proitsi et al. showed no association between a genetic risk score of 157 lipid SNPs weighted by their HDL-C effect and AD in up to 10,578 individuals [17]. While Proitsi et al. used a genetic risk score of all genome-wide associated SNPs, we focused on one mechanism of raising HDL-C, through the gene *CETP*.

The strengths of our study include the large sample sizes that were used for the summary statistics allowing for precise estimates of effect and the multiple statistical analyses pointing to the same conclusion. Despite the large samples and multiple methods, limitations of our study are as follows. First, we had power to detect odds ratios >1.18 or <0.85 for genetically increased HDL-C on risk of AD. If there is a smaller effect of HDL-C on AD, we may not have been able to detect it. Another study showed that variation in *CETP* associated with higher HDL-C is also associated with an increased risk of intracerebral hemorrhage [18]. We used the same set of SNPs. Second, *CETP* SNPs are also known to be associated with other lipid levels and therefore the other lipid fractions (i.e., lower low-density lipoprotein cholesterol or triglycerides) may hide a true relationship between HDL-C and AD. Third, our study focused on the effects of HDL-C in the population, and we were not able to determine whether extreme HDL-C levels through *CETP* have an association with AD. Fourth, other factors that influence HDL-C levels may have a causal effect on AD, and these cannot be elucidated by the present study. Fifth, the studies that contributed to the results were predominately of European origin and therefore we cannot generalize these results to other ancestries.

Despite the promise of high HDL-C providing protection for AD, we do not find evidence that increasing HDL-C through the *CETP* will result in lower risk for AD.

**Research in Context**

1. Systematic review: The connection between cholesterol levels and risk of Alzheimer's disease (AD) and dementia has been suggested as a promising avenue for risk prediction as well as risk reduction. There is conflicting evidence whether high-density lipoprotein cholesterol (HDL-C) levels are a risk factor for AD and dementia.
2. Interpretation: We performed a study with large sample sizes to look at whether genetically predicted HDL-C through cholesteryl ester transfer protein is associated with risk of AD. We found that single nucleotide polymorphisms in the cholesteryl ester transfer protein locus with a large effect on HDL-C were not associated with risk of AD, and that genetically predicted HDL-C through cholesteryl ester transfer protein did not associate with risk of AD, suggesting that high HDL-C does not provide protection for AD.
3. Future directions: This study does not rule out other mechanisms by which HDL-C affects risk of AD; these should be explored.

## Supplementary data

Supplementary Figure 1 and Collaborators from the IGAP
