# Supplementary material for: Genetically elevated high-density lipoprotein cholesterol through the cholesteryl ester transfer protein gene does not associate with risk of Alzheimer's disease
Source: Alzheimers Dement (Amst). 2018 Sep 22;10:595–8. doi: 10.1016/j.dadm.2018.08.008 (PMC6215982; doi:10.1016/j.dadm.2018.08.008)
Supplement: Supplementary Figure 1 and Collaborators from the IGAP [file mmc1.docx]

**Supplemental Figure 1. Relationships between effect sizes.**

A). Scatter plot of AD effect sizes (beta) from model 1 (PC adjustment only) and model 2 (PC plus age and sex) for 10 SNPs

B). Scatter plot of AD effect sizes from model 1 and model 2 after removing SNP with highly discordant effects between adjustment schemes.

C). Scatter plot of HDL effect size against AD effect size from model 1.

D). Scatter plot of HDL effect size against AD effect size from model 2.

HDL-C was measured in mg/dl and inverse normalized prior to associating to the SNPs. Therefore, the effect sizes for HDL-C against each SNP are in standard deviation units. HDL-C has a standard deviation of ~14 mg/dl in the population.

**Collaborators from the International Genomics of Alzheimer’s Project (IGAP):** Sims R^1^, van der Lee SJ^2^, Naj AC^3^, Bellenguez C^4,5,6^, Badarinarayan N^1^, Jakobsdottir J^7^, Kunkle BW^8^, Boland A^9^, Raybould R^1^, Bis JC^10^, Martin ER^8,11^, Grenier-Boley B^4,5,6^, Heilmann-Heimbach S^12,13^, Chouraki V^14,15^, Kuzma AB^16^, Sleegers K^17,18^, Vronskaya M^1^, Ruiz A^19^, Graham RR^20^, Olaso R^9^, Hoffmann P^12,13,21^, Grove ML^22^, Vardarajan BN^23,24,25^, Hiltunen M^26,27^, Nöthen MM^12,13^, White CC^28^, Hamilton-Nelson KL^8^, Epelbaum J^29^, Maier W^30,31^, Choi SH^14,32^, Beecham GW^8,11^, Dulary C^9^, Herms S^12,13,21^, Smith AV^7,33^, Funk CC^34^, Derbois C^9^, Forstner AJ^12,13^, Ahmad S^2^, Li H^34^, Bacq D^9^, Harold D^35^, Satizabal CL^14,15^, Valladares O^16^, Squassina A^36^, Thomas R^1^, Brody JA^10^, Qu L^16^, Sánchez-Juan P^37^, Morgan T^1^, Wolters FJ^2^, Zhao Y^16^, Garcia FS^38^, Denning N^1^, Fornage M^39^, Malamon J^16^, Naranjo MCD^38^, Majounie E^1^, Mosley TH^40^, Dombroski B^16^, Wallon D^41,42^, Lupton MK^43,44^, Dupuis J^32^, Whitehead P^8^, Fratiglioni L^45,46^, Medway C^47^, Jian X^39^, Mukherjee S^48^, Keller L^46^, Brown K^47^, Lin H^49^, Cantwell LB^16^, Panza F^50^, McGuinness B^51^, Moreno-Grau S^19^, Burgess JD^52^, Solfrizzi V^53^, Proitsi P^43^, Adams HH^2^, Allen M^52^, Seripa D^54^, Pastor P^55^, Cupples LA^15,32^, Price ND^34^, Hannequin D^42,56^, Frank-García A^57,58,59^, Levy D^14,15,60^, Chakrabarty P^61^, Caffarra P^62,63^, Giegling I^64^, Beiser AS^15,32^, Giedraitis V^65^, Hampel H^66,67,68^, Garcia ME^69^, Wang X^52^, Lannfelt L^65^, Mecocci P^55^, Eiriksdottir G^7^, Crane PK^48^, Pasquier F^70,71^, Boccardi V^55^, Henández I^19^, Barber RC^72^, Scherer M^73^, Tarraga L^19^, Adams PM^74^, Leber M^75^, Chen Y^32^, Albert MS^76^, Riedel-Heller S^77^, Emilsson V^7,78^, Beekly D^79^, Braae A^80^, Schmidt R^81^, Blacker D^82,83^, Masullo C^84^, Schmidt H^85^, Doody RS^86^, Spalletta G^87^, Longstreth WT Jr^88,89^, Fairchild TJ^90^, Bossù P^87^, Lopez OL^91,92^, Frosch MP^93^, Sacchinelli E^87^, Ghetti B^94^, Yang Q^32^, Huebinger RM^95^, Jessen F^30,31,75^, Li S^32^, Kamboh MI^96,97^, Morris J^98,99^, Sotolongo-Grau O^19^, Katz MJ^100^, Corcoran C^101^, Dunstan M^1^, Braddel A^1^, Thomas C^1^, Meggy A^1^, Marshall R^1^, Gerrish A^1^, Chapman J^1^, Aguilar M^102,103^, Taylor S^1^, Hill M^1^, Fairén MD^59,102^, Hodges A^104^, Vellas B^105^, Soininen H^27^, Kloszewska I^106^, Daniilidou M^107^, Uphill J^108^, Patel Y^43^, Hughes JT^43^, Lord J^47^, Turton J^47^, Hartmann AM^64^, Cecchetti R^55^, Fenoglio C^109^, Serpente M^109^, Arcaro M^109^, Caltagirone C^87^, Orfei MD^87^, Ciaramella A^87^, Pichler S^110^, Mayhaus M^110^, Gu W^110^, Lleó A^59,111^, Fortea J^59,111^, Blesa R^59,111^, Barber IS^80^, Brookes K^80^, Cupidi C^112^, Maletta RG^112^, Carrell D^113^, Sorbi S^114,115^, Moebus S^116^, Urbano M^54^, Pilotto A^54^, Kornhuber J^117^, Bosco P^118^, Todd S^51^, Craig D^51^, Johnston J^51^, Gill M^119^, Lawlor B^119^, Lynch A^119^, Fox NC^120^, Hardy J^120^; ARUK Consortium, Albin RL^121,122,123^, Apostolova LG^124,125,126,127^, Arnold SE^128^, Asthana S^129,130,131^, Atwood CS^129,130,131^, Baldwin CT^132^, Barnes LL^133,134,135^, Barral S^23,24,25^, Beach TG^136^, Becker JT^137^, Bigio EH^138,139^, Bird TD^88,140^, Boeve BF^141^, Bowen JD^142^, Boxer A^143^, Burke JR^144^, Burns JM^145^, Buxbaum JD^146,147,148^, Cairns NJ^149^, Cao C^150^, Carlson CS^151^, Carlsson CM^130,131^, Carney RM^152^, Carrasquillo MM^52^, Carroll SL^153^, Diaz CC^61^, Chui HC^154^, Clark DG^155,156^, Cribbs DH^157^, Crocco EA^152^, DeCarli C^158^, Dick M^159^, Duara R^160^, Evans DA^161^, Faber KM^125^, Fallon KB^162^, Fardo DW^163^, Farlow MR^126^, Ferris S^164^, Foroud TM^125^, Galasko DR^165^, Gearing M^166,167^, Geschwind DH^168^, Gilbert JR^8,11^, Graff-Radford NR^52,169^, Green RC^170^, Growdon JH^171^, Hamilton RL^172^, Harrell LE^173^, Honig LS^23^, Huentelman MJ^174^, Hulette CM^175^, Hyman BT^171^, Jarvik GP^176,177^, Abner E^178^, Jin LW^179^, Jun G^132,180,181^, Karydas A^143^, Kaye JA^182,183^, Kim R^184^, Kowall NW^185,186^, Kramer JH^187^, LaFerla FM^188^, Lah JJ^189^, Leverenz JB^190^, Levey AI^189^, Li G^113,140^, Lieberman AP^191^, Lunetta KL^180^, Lyketsos CG^192^, Marson DC^173^, Martiniuk F^193^, Mash DC^194^, Masliah E^165,195^, McCormick WC^48^, McCurry SM^196^, McDavid AN^151^, McKee AC^185,186^, Mesulam M^139,197^, Miller BL^143^, Miller CA^198^, Miller JW^179^, Morris JC^149,199^, Murrell JR^94,125^, Myers AJ^152^, O'Bryant S^200^, Olichney JM^158^, Pankratz VS^201^, Parisi JE^202^, Paulson HL^121,123^, Perry W^8^, Peskind E^113^, Pierce A^157^, Poon WW^159^, Potter H^203^, Quinn JF^182,183^, Raj A^150^, Raskind M^113^, Reisberg B^164,204^, Reitz C^24,25,205^, Ringman JM^206^, Roberson ED^173^, Rogaeva E^207^, Rosen HJ^143^, Rosenberg RN^208^, Sager MA^130^, Saykin AJ^125,127^, Schneider JA^133,135,209^, Schneider LS^154,210^, Seeley WW^143^, Smith AG^150^, Sonnen JA^211^, Spina S^94^, Stern RA^185^, Swerdlow RH^145^, Tanzi RE^171^, Thornton-Wells TA^212^, Trojanowski JQ^213^, Troncoso JC^214^, Van Deerlin VM^213^, Van Eldik LJ^215^, Vinters HV^206,216^, Vonsattel JP^217^, Weintraub S^139,218^, Welsh-Bohmer KA^144,219^, Wilhelmsen KC^220^, Williamson J^23^, Wingo TS^189^, Woltjer RL^221^, Wright CB^222^, Yu CE^48^, Yu L^133,135^, Garzia F^9^, Golamaully F^9^, Septier G^9^, Engelborghs S^18,223^, Vandenberghe R^223,224^, De Deyn PP^18,223^, Fernadez CM^38^, Benito YA^38^, Thonberg H^225,226^, Forsell C^225,226^, Lilius L^225,226^, Kinhult-Stählbom A^225,226^, Kilander L^65^, Brundin R^65^, Concari L^62,63^, Helisalmi S^27,227^, Koivisto AM^27,227^, Haapasalo A^27,227^, Dermecourt V^228^, Fievet N^4,5,6,229^, Hanon O^229^, Dufouil C^230,231^, Brice A^232,233^, Ritchie K^234^, Dubois B^235,236,237,238^, Himali JJ^14^, Keene CD^211^, Tschanz J^101^, Fitzpatrick AL^89,239^, Kukull WA^89^, Norton M^101^, Aspelund T^7,240^, Larson EB^48,241^, Munger R^101^, Rotter JI^242^, Lipton RB^100^, Bullido MJ^58,59,243^, Hofman A^2^, Montine TJ^211^, Coto E^244^, Boerwinkle E^22,245^, Petersen RC^141^, Alvarez V^244^, Rivadeneira F^2,246,247^, Reiman EM^174,248,249,250^, Gallo M^112^, O'Donnell CJ^15^, Reisch JS^251^, Bruni AC^112^, Royall DR^252^, Dichgans M^253,254^, Sano M^148^, Galimberti D^109^, St George-Hyslop P^207,255^, Scarpini E^109^, Tsuang DW^113,140^, Mancuso M^256^, Bonuccelli U^256^, Winslow AR^257^, Daniele A^258^, Wu CK^259^; GERAD/PERADES, CHARGE, ADGC, EADI, Peters O^260^, Nacmias B^114,115^, Riemenschneider M^110^, Heun R^31^, Brayne C^261^, Rubinsztein DC^255^, Bras J^120,262^, Guerreiro R^120,262^, Al-Chalabi A^263^, Shaw CE^263^, Collinge J^108^, Mann D^264^, Tsolaki M^265^, Clarimón J^59,111^, Sussams R^266^, Lovestone S^267^, O'Donovan MC^1^, Owen MJ^1^, Behrens TW^20^, Mead S^108^, Goate AM^147^, Uitterlinden AG^2,108,109^, Holmes C^140^, Cruchaga C^98,99^, Ingelsson M^65^, Bennett DA^133,135^, Powell J^43^, Golde TE^61,268^, Graff C^45,226^, De Jager PL^269^, Morgan K^47^, Ertekin-Taner N^52,169^, Combarros O^37^, Psaty BM^10,59,89,270^, Passmore P^51^, Younkin SG^52,169^, Berr C^235,271,272^, Gudnason V^7,33^, Rujescu D^64^, Dickson DW^52^, Dartigues JF^273^, DeStefano AL^15,32^, Ortega-Cubero S^59,274,275^, Hakonarson H^276^, Campion D^41,42^, Boada M^19^, Kauwe JK^277^, Farrer LA^14,132,180,181,185^, Van Broeckhoven C^17,18^, Ikram MA^2,278^, Jones L^1^, Haines JL^279^, Tzourio C^232,280^, Launer LJ^69^, Escott-Price V^1^, Mayeux R^23,24,25^, Deleuze JF^9^, Amin N^2^, Holmans PA^1^, Pericak-Vance MA^8,11^, Amouyel P^4,5,6,70^, van Duijn CM^2^, Ramirez A^12,31,75^, Wang LS^16^, Lambert JC^4,5,6^, Seshadri S^14,15^, Williams J^1^, Schellenberg GD^16^.

1 Institute of Psychological Medicine and Clinical Neurosciences, MRC Centre for Neuropsychiatric Genetics and Genomics, Cardiff University, Cardiff, UK.

2 Department of Epidemiology, Erasmus Medical Center, Rotterdam, the Netherlands.

3 Department of Biostatistics and Epidemiology/Center for Clinical Epidemiology and Biostatistics, University of Pennsylvania Perelman School of Medicine, Philadelphia, Pennsylvania, USA.

4 INSERM, U1167, RID-AGE-Risk Factors and Molecular Determinants of Aging-Related Diseases, Lille, France.

5 Institut Pasteur de Lille, Lille, France.

6 University Lille, U1167-Excellence Laboratory LabEx DISTALZ, Lille, France.

7 Icelandic Heart Association, Kopavogur, Iceland.

8 John P. Hussman Institute for Human Genomics, University of Miami, Miami, Florida, USA.

9 CEA/Institut de Génomique, Centre National de Génotypage, Evry, France.

10 Cardiovascular Health Research Unit, Department of Medicine, University of Washington, Seattle, Washington, USA.

11 Dr. John T. Macdonald Foundation, Department of Human Genetics, University of Miami, Miami, Florida, USA.

12 Institute of Human Genetics, University of Bonn, Bonn, Germany.

13 Department of Genomics, Life &Brain Center, University of Bonn, Bonn, Germany.

14 Boston University School of Medicine, Boston, Massachusetts, USA.

15 Framingham Heart Study, Framingham, Massachusetts, USA.

16 Penn Neurodegeneration Genomics Center, Department of Pathology and Laboratory Medicine, University of Pennsylvania Perelman School of Medicine, Philadelphia, Pennsylvania, USA.

17 Neurodegenerative Brain Diseases Group, Department of Molecular Genetics, VIB, Antwerp, Belgium.

18 Institute Born-Bunge, University of Antwerp, Antwerp, Belgium.

19 Research Center and Memory Clinic of Fundació ACE, Institut Català de Neurociències Aplicades, Barcelona, Spain.

20 Immunology Biomarkers Group, Genentech, South San Francisco, California, USA.

21 Division of Medical Genetics, University Hospital and Department of Biomedicine, University of Basel, Basel, Switzerland.

22 School of Public Health, Human Genetics Center, University of Texas Health Science Center at Houston, Houston, Texas, USA.

23 Taub Institute on Alzheimer's Disease and the Aging Brain, Department of Neurology, Columbia University, New York, New York, USA.

24 Gertrude H. Sergievsky Center, Columbia University, New York, New York, USA.

25 Department of Neurology, Columbia University, New York, New York, USA.

26 Institute of Biomedicine, University of Eastern Finland, Kuopio, Finland.

27 Department of Neurology, Kuopio University Hospital, Kuopio, Finland.

28 Program in Translational NeuroPsychiatric Genomics, Institute for the Neurosciences, Departments of Neurology and Psychiatry, Brigham and Women's Hospital, Boston, Massachusetts, USA.

29 UMR 894, Center for Psychiatry and Neuroscience, INSERM, Université Paris Descartes, Paris, France.

30 German Center for Neurodegenerative Diseases (DZNE), Bonn, Germany.

31 Department of Psychiatry and Psychotherapy, University of Bonn, Bonn, Germany.

32 Department of Biostatistics, Boston University School of Public Health, Boston, Massachusetts, USA.

33 Faculty of Medicine, University of Iceland, Reykjavik, Iceland.

34 Institute for Systems Biology, Seattle, Washington, USA.

35 School of Biotechnology, Dublin City University, Dublin, Ireland.

36 Section of Neuroscience and Clinical Pharmacology, Department of Biomedical Sciences, University of Cagliari, Cagliari, Italy.

37 Neurology Service and CIBERNED, 'Marqués de Valdecilla' University Hospital (University of Cantabria and IFIMAV), Santander, Spain.

38 Department of Immunology, Hospital Universitario Doctor Negrín, Las Palmas de Gran Canaria, Spain.

39 Brown Foundation Institute of Molecular Medicine, University of Texas Health Sciences Center at Houston, Houston, Texas, USA.

40 Departments of Medicine, Geriatrics, Gerontology and Neurology, University of Mississippi Medical Center, Jackson, Mississippi, USA.

41 Centre Hospitalier du Rouvray, Sotteville les Rouen, France.

42 INSERM U1079, Rouen University, IRIB, Normandy University, Rouen, France.

43 Department of Basic and Clinical Neuroscience, Institute of Psychiatry, Psychology and Neuroscience, King's College London, London, UK.

44 Genetic Epidemiology, QIMR Berghofer Medical Research Institute, Herston, Queensland, Australia.

45 Department of Geriatric Medicine, Karolinska University Hospital Huddinge, Stockholm, Sweden.

46 Aging Research Center, Department of Neurobiology, Care Sciences and Society, Karolinska Institutet and Stockholm University, Stockholm, Sweden.

47 Institute of Genetics, Queen's Medical Centre, University of Nottingham, Nottingham, UK.

48 Department of Medicine, University of Washington, Seattle, Washington, USA.

49 Section of Computational Biomedicine, Department of Medicine, Boston University School of Medicine, Boston, Massachusetts, USA.

50 Neurodegenerative Disease Unit, Department of Basic Medicine, Neuroscience, and Sense Organs, University of Bari Aldo Moro, Bari, Italy.

51 Centre for Public Health, School of Medicine, Dentistry and Biomedical Sciences, Queen's University, Belfast, UK.

52 Department of Neuroscience, Mayo Clinic, Jacksonville, Florida, USA.

53 Geriatric Medicine-Memory Unit and Rare Disease Centre, University of Bari Aldo Moro, Bari, Italy.

54 Geriatric Unit and Gerontology-Geriatrics Research Laboratory, Department of Medical Sciences, IRCCS Casa Sollievo della Sofferenza, San Giovanni Rotondo, Italy.

55 Section of Gerontology and Geriatrics, Department of Medicine, University of Perugia, Perugia, Italy.

56 Department of Neurology, Rouen University Hospital, Rouen, France.

57 Department of Neurology, University Hospital La Paz, Universidad Autónoma de Madrid, Madrid, Spain.

58 Instituto de Investigación Sanitaria Hospital la Paz (IdiPAZ), Madrid, Spain.

59 Centro de Investigación Biomédica en Red de Enfermedades Neurodegenerativas (CIBERNED), Instituto de Salud Carlos III, Madrid, Spain.

60 National Heart, Lung, and Blood Institute, Bethesda, Maryland, USA.

61 Center for Translational Research in Neurodegenerative Disease, Department of Neuroscience, University of Florida, Gainesville, Florida, USA.

62 Department of Neuroscience, University of Parma, Parma, Italy.

63 Center for Cognitive Disorders AUSL, Parma, Italy.

64 Department of Psychiatry, Martin Luther University Halle-Wittenberg, Halle, Germany.

65 Department of Public Health/Geriatrics, Uppsala University, Uppsala, Sweden.

66 AXA Research Fund and UPMC Chair, Paris, France.

67 Sorbonne Universités, Université Pierre et Marie Curie, Paris, France.

68 Institut de la Mémoire et de la Maladie d'Alzheimer (IM2A) and Institut du Cerveau et de la Moelle Épinière (ICM), Département de Neurologie, Hôpital de la Pitié-Salpêtrière, Paris, France.

69 Laboratory of Epidemiology and Population Sciences, National Institute on Aging, Bethesda, Maryland, USA.

70 Centre Hospitalier Universitaire de Lille, Epidemiology and Public Health Department, Lille, France.

71 INSERM UMRS 1171, CNR-Maj, Lille, France.

72 Department of Pharmacology and Neuroscience, University of North Texas Health Science Center, Fort Worth, Texas, USA.

73 Department of Primary Medical Care, University Medical Centre Hamburg-Eppendorf, Hamburg, Germany.

74 Department of Psychiatry, University of Texas Southwestern Medical Center, Dallas, Texas, USA.

75 Department of Psychiatry and Psychotherapy, University of Cologne, Cologne, Germany.

76 Department of Neurology, Johns Hopkins University, Baltimore, Maryland, USA.

77 Institute of Social Medicine, Occupational Health and Public Health, University of Leipzig, Leipzig, Germany.

78 Faculty of Pharmaceutical Sciences, University of Iceland, Reykjavik, Iceland.

79 National Alzheimer's Coordinating Center, University of Washington, Seattle, Washington, USA.

80 Schools of Life Sciences and Medicine, University of Nottingham, Nottingham, UK.

81 Department of Neurology, Clinical Division of Neurogeriatrics, Medical University Graz, Graz, Austria.

82 Department of Epidemiology, Harvard School of Public Health, Boston, Massachusetts, USA.

83 Department of Psychiatry, Massachusetts General Hospital/Harvard Medical School, Boston, Massachusetts, USA.

84 Department of Neurology, Catholic University of Rome, Rome, Italy.

85 Institute of Molecular Biology and Biochemistry, Medical University Graz, Graz, Austria.

86 Alzheimer's Disease and Memory Disorders Center, Baylor College of Medicine, Houston, Texas, USA.

87 Experimental Neuropsychiatry Laboratory, IRCCS Santa Lucia Foundation, Department of Clinical and Behavioural Neurology, Rome, Italy.

88 Department of Neurology, University of Washington, Seattle, Washington, USA.

89 Department of Epidemiology, University of Washington, Seattle, Washington, USA.

90 Office of Strategy and Measurement, University of North Texas Health Science Center, Fort Worth, Texas, USA.

91 Department of Psychiatry, University of Pittsburgh, Pittsburgh, Pennsylvania, USA.

92 Department of Neurology, University of Pittsburgh, Pittsburgh, Pennsylvania, USA.

93 C.S. Kubik Laboratory for Neuropathology, Massachusetts General Hospital, Charlestown, Massachusetts, USA.

94 Department of Pathology and Laboratory Medicine, Indiana University, Indianapolis, Indiana, USA.

95 Department of Surgery, University of Texas Southwestern Medical Center, Dallas, Texas, USA.

96 Alzheimer's Disease Research Center, University of Pittsburgh, Pittsburgh, Pennsylvania, USA.

97 Department of Human Genetics, University of Pittsburgh, Pittsburgh, Pennsylvania, USA.

98 Department of Psychiatry, Washington University School of Medicine, St. Louis, Missouri, USA.

99 Hope Center Program on Protein Aggregation and Neurodegeneration, Washington University School of Medicine, St. Louis, Missouri, USA.

100 Department of Neurology, Albert Einstein College of Medicine, Bronx, New York, USA.

101 Department of Mathematics and Statistics, Utah State University, Logan, Utah, USA.

102 Fundació per la Recerca Biomèdica i Social Mútua Terrassa, Terrassa, Barcelona, Spain.

103 Memory Unit, Department of Neurology, Hospital Universitario Mútua Terrassa, Terrassa, Barcelona, Spain.

104 Department of Old Age Psychiatry, Institute of Psychiatry, Psychology and Neuroscience, King's College London, London, UK.

105 INSERM U558, University of Toulouse, Toulouse, France.

106 Elderly and Psychiatric Disorders Department, Medical University of Lodz, Lodz, Poland.

107 Department of Health Sciences, Psychiatry for the Elderly, University of Leicester, Leicester, UK.

108 Department of Neurodegenerative Disease, MRC Prion Unit, UCL Institute of Neurology, London, UK.

109 Department of Pathophysiology and Transplantation, University of Milan, Fondazione Ca' Granda, IRCCS Ospedale Policlinico, Milan, Italy.

110 Department of Psychiatry and Psychotherapy, University Hospital, Saarland, Germany.

111 Memory Unit, Neurology Department and Sant Pau Biomedical Research Institute, Hospital de la Santa Creu i Sant Pau, Autonomous University of Barcelona, Barcelona, Spain.

112 Regional Neurogenetic Centre (CRN), ASP Catanzaro, Lamezia Terme, Italy.

113 Department of Psychiatry and Behavioral Sciences, University of Washington School of Medicine, Seattle, Washington, USA.

114 NEUROFARBA (Department of Neuroscience, Psychology, Drug Research and Child Health), University of Florence, Florence, Italy.

115 IRCCS 'Don Carlo Gnocchi', Florence, Italy.

116 Institute for Medical Informatics, Biometry and Epidemiology, University Hospital of Essen, University Duisburg-Essen, Essen, Germany.

117 Department of Psychiatry and Psychotherapy, University of Erlangen-Nuremberg, Erlangen, Germany.

118 Istituto di Ricovero e Cura a Carattere Scientifico (IRCCS) Associazione Oasi Maria Santissima Srl, Troina, Italy.

119 Mercers Institute for Research on Aging, St. James Hospital and Trinity College, Dublin, Ireland.

120 Department of Molecular Neuroscience, UCL, Institute of Neurology, London, UK.

121 Department of Neurology, University of Michigan, Ann Arbor, Michigan, USA.

122 Geriatric Research, Education and Clinical Center (GRECC), VA Ann Arbor Healthcare System (VAAAHS), Ann Arbor, Michigan, USA.

123 Michigan Alzheimer Disease Center, Ann Arbor, Michigan, USA.

124 Indiana Alzheimer's Disease Center, Indiana University School of Medicine, Indianapolis, Indiana, USA.

125 Department of Medical and Molecular Genetics, Indiana University, Indianapolis, Indiana, USA.

126 Department of Neurology, Indiana University, Indianapolis, Indiana, USA.

127 Department of Radiology and Imaging Sciences, Indiana University, Indianapolis, Indiana, USA.

128 Department of Psychiatry, University of Pennsylvania Perelman School of Medicine, Philadelphia, Pennsylvania, USA.

129 Geriatric Research, Education and Clinical Center (GRECC), University of Wisconsin, Madison, Wisconsin, USA.

130 Department of Medicine, University of Wisconsin, Madison, Wisconsin, USA.

131 Wisconsin Alzheimer's Disease Research Center, Madison, Wisconsin, USA.

132 Department of Medicine (Genetics Program), Boston University, Boston, Massachusetts, USA.

133 Department of Neurological Sciences, Rush University Medical Center, Chicago, Illinois, USA.

134 Department of Behavioral Sciences, Rush University Medical Center, Chicago, Illinois, USA.

135 Rush Alzheimer's Disease Center, Rush University Medical Center, Chicago, Illinois, USA.

136 Civin Laboratory for Neuropathology, Banner Sun Health Research Institute, Phoenix, Arizona, USA.

137 Departments of Psychiatry, Neurology, and Psychology, University of Pittsburgh School of Medicine, Pittsburgh, Pennsylvania, USA.

138 Department of Pathology, Northwestern University Feinberg School of Medicine, Chicago, Illinois, USA.

139 Cognitive Neurology and Alzheimer's Disease Center, Northwestern University Feinberg School of Medicine, Chicago, Illinois, USA.

140 VA Puget Sound Health Care System/GRECC, Seattle, Washington, USA.

141 Department of Neurology, Mayo Clinic, Rochester, Minnesota, USA.

142 Swedish Medical Center, Seattle, Washington, USA.

143 Department of Neurology, University of California, San Francisco, San Francisco, California, USA.

144 Department of Medicine, Duke University, Durham, North Carolina, USA.

145 University of Kansas Alzheimer's Disease Center, University of Kansas Medical Center, Kansas City, Kansas, USA.

146 Department of Genetics and Genomic Sciences, Mount Sinai School of Medicine, New York, New York, USA.

147 Department of Neuroscience, Mount Sinai School of Medicine, New York, New York, USA.

148 Department of Psychiatry, Mount Sinai School of Medicine, New York, New York, USA.

149 Department of Pathology and Immunology, Washington University, St. Louis, Missouri, USA.

150 USF Health Byrd Alzheimer's Institute, University of South Florida, Tampa, Florida, USA.

151 Fred Hutchinson Cancer Research Center, Seattle, Washington, USA.

152 Department of Psychiatry and Behavioral Sciences, Miller School of Medicine, University of Miami, Miami, Florida, USA.

153 Department of Pathology and Laboratory Medicine, Medical University of South Carolina, Charleston, South Carolina, USA.

154 Department of Neurology, University of Southern California, Los Angeles, California, USA.

155 Department of Neurology, Medical University of South Carolina, Charleston, South Carolina, USA.

156 Department of Neurology, Ralph H. Johnson VA Medical Center, Charleston, South Carolina, USA.

157 Department of Neurology, University of California, Irvine, Irvine, California, USA.

158 Department of Neurology, University of California, Davis, Sacramento, California, USA.

159 Institute for Memory Impairments and Neurological Disorders, University of California, Irvine, Irvine, California, USA.

160 Wien Center for Alzheimer's Disease and Memory Disorders, Mount Sinai Medical Center, Miami Beach, Florida, USA.

161 Rush Institute for Healthy Aging, Department of Internal Medicine, Rush University Medical Center, Chicago, Illinois, USA.

162 Department of Pathology, University of Alabama at Birmingham, Birmingham, Alabama, USA.

163 Sanders-Brown Center on Aging, Department of Biostatistics, University of Kentucky, Lexington, Kentucky, USA.

164 Department of Psychiatry, New York University, New York, New York, USA.

165 Department of Neurosciences, University of California, San Diego, La Jolla, California, USA.

166 Department of Pathology and Laboratory Medicine, Emory University, Atlanta, Georgia, USA.

167 Emory Alzheimer's Disease Center, Emory University, Atlanta, Georgia, USA.

168 Neurogenetics Program, University of California, Los Angeles, Los Angeles, California, USA.

169 Department of Neurology, Mayo Clinic, Jacksonville, Florida, USA.

170 Division of Genetics, Department of Medicine and Partners Center for Personalized Genetic Medicine, Brigham and Women's Hospital and Harvard Medical School, Boston, Massachusetts, USA.

171 Department of Neurology, Massachusetts General Hospital/Harvard Medical School, Boston, Massachusetts, USA.

172 Department of Pathology (Neuropathology), University of Pittsburgh, Pittsburgh, Pennsylvania, USA.

173 Department of Neurology, University of Alabama at Birmingham, Birmingham, Alabama, USA.

174 Neurogenomics Division, Translational Genomics Research Institute, Phoenix, Arizona, USA.

175 Department of Pathology, Duke University, Durham, North Carolina, USA.

176 Department of Genome Sciences, University of Washington, Seattle, Washington, USA.

177 Department of Medicine (Medical Genetics), University of Washington, Seattle, Washington, USA.

178 Sanders-Brown Center on Aging, College of Public Health, Department of Epidemiology, University of Kentucky, Lexington, Kentucky, USA.

179 Department of Pathology and Laboratory Medicine, University of California, Davis, Sacramento, California, USA.

180 Department of Biostatistics, Boston University, Boston, Massachusetts, USA.

181 Department of Ophthalmology, Boston University, Boston, Massachusetts, USA.

182 Department of Neurology, Oregon Health &Science University, Portland, Oregon, USA.

183 Department of Neurology, Portland Veterans Affairs Medical Center, Portland, Oregon, USA.

184 Department of Pathology and Laboratory Medicine, University of California, Irvine, Irvine, California, USA.

185 Department of Neurology, Boston University, Boston, Massachusetts, USA.

186 Department of Pathology, Boston University, Boston, Massachusetts, USA.

187 Department of Neuropsychology, University of California, San Francisco, San Francisco, California, USA.

188 Department of Neurobiology and Behavior, University of California, Irvine, Irvine, California, USA.

189 Department of Neurology, Emory University, Atlanta, Georgia, USA.

190 Cleveland Clinic Lou Ruvo Center for Brain Health, Cleveland Clinic, Cleveland, Ohio, USA.

191 Department of Pathology, University of Michigan, Ann Arbor, Michigan, USA.

192 Department of Psychiatry, Johns Hopkins University, Baltimore, Maryland, USA.

193 Department of Medicine-Pulmonary, New York University, New York, New York, USA.

194 Department of Neurology, University of Miami, Miami, Florida, USA.

195 Department of Pathology, University of California, San Diego, La Jolla, California, USA.

196 School of Nursing Northwest Research Group on Aging, University of Washington, Seattle, Washington, USA.

197 Department of Neurology, Northwestern University Feinberg School of Medicine, Chicago, Illinois, USA.

198 Department of Pathology, University of Southern California, Los Angeles, California, USA.

199 Department of Neurology, Washington University, St. Louis, Missouri, USA.

200 Internal Medicine, Division of Geriatrics, University of North Texas Health Science Center, Fort Worth, Texas, USA.

201 Department of Internal Medicine, University of New Mexico Health Sciences Center, Albuquerque, New Mexico, USA.

202 Department of Laboratory Medicine and Pathology, Mayo Clinic, Rochester, Minnesota, USA.

203 Department of Neurology, University of Colorado School of Medicine, Aurora, Colorado, USA.

204 Alzheimer's Disease Center, New York University, New York, New York, USA.

205 Department of Epidemiology, Columbia University, New York, New York, USA.

206 Department of Neurology, University of California, Los Angeles, Los Angeles, California, USA.

207 Tanz Centre for Research in Neurodegenerative Disease, University of Toronto, Toronto, Ontario, Canada.

208 Department of Neurology, University of Texas Southwestern Medical Center, Dallas, Texas, USA.

209 Department of Pathology (Neuropathology), Rush University Medical Center, Chicago, Illinois, USA.

210 Department of Psychiatry, University of Southern California, Los Angeles, California, USA.

211 Department of Pathology, University of Washington, Seattle, Washington, USA.

212 Translational Medicine, Novartis Institutes for Biomedical Research, Cambridge, Massachusetts, USA.

213 Department of Pathology and Laboratory Medicine, University of Pennsylvania Perelman School of Medicine, Philadelphia, Pennsylvania, USA.

214 Department of Pathology, Johns Hopkins University, Baltimore, Maryland, USA.

215 Sanders-Brown Center on Aging, Department of Anatomy and Neurobiology, University of Kentucky, Lexington, Kentucky, USA.

216 Department of Pathology and Laboratory Medicine, University of California, Los Angeles, Los Angeles, California, USA.

217 Taub Institute on Alzheimer's Disease and the Aging Brain, Department of Pathology, Columbia University, New York, New York, USA.

218 Department of Psychiatry, Northwestern University Feinberg School of Medicine, Chicago, Illinois, USA.

219 Department of Psychiatry and Behavioral Sciences, Duke University, Durham, North Carolina, USA.

220 Department of Genetics, University of North Carolina at Chapel Hill, Chapel Hill, North Carolina, USA.

221 Department of Pathology, Oregon Health &Science University, Portland, Oregon, USA.

222 Evelyn F. McKnight Brain Institute, Department of Neurology, Miller School of Medicine, University of Miami, Miami, Florida, USA.

223 Department of Neurology and Memory Clinic, Hospital Network Antwerp, Antwerp, Belgium.

224 Laboratory for Cognitive Neurology, Department of Neurology, University of Leuven, Leuven, Belgium.

225 Department of Geriatric Medicine, Genetics Unit, Karolinska University Hospital Huddinge, Stockholm, Sweden.

226 Department of Neurobiology, Care Sciences and Society, KIADRC, Karolinska Institutet, Stockholm, Sweden.

227 Institute of Clinical Medicine/Neurology, University of Eastern Finland, Kuopio, Finland.

228 CHU Lille, Memory Center of Lille (Centre Mémoire de Ressources et de Recherche), Lille, France.

229 University Paris Descartes, EA 4468, AP-HP, Hôpital Broca, Geriatrics Department, Paris, France.

230 University of Bordeaux, Neuroepidemiology, Bordeaux, France.

231 INSERM, Neuroepidemiology, UMR 897, Bordeaux, France.

232 INSERM U1127, CNRS UMR 7225, Sorbonne Universités, UPMC Université Paris 06, UMRS 1127, Institut du Cerveau et de la Moelle Épinière, Paris, France.

233 AP-HP, Department of Genetics, Pitié-Salpêtrière Hospital, Paris, France.

234 INSERM U1061, La Colombière Hospital, Montpellier, France.

235 Institut de la Mémoire et de la Maladie d'Alzheimer (IM2A), Département de Neurologie, Hôpital de la Pitié-Salpêtrière, AP-HP, Paris, France.

236 Institut des Neurosciences Translationnelles de Paris (IHU-A-ICM), Institut du Cerveau et de la Moelle Épinière (ICM), Paris, France.

237 INSERM, CNRS, UMRS 975, Institut du Cerveau et de la Moelle Épinière (ICM), Paris, France.

238 Sorbonne Universités, Université Pierre et Marie Curie, Hôpital de la Pitié-Salpêtrière, AP-HP, Paris, France.

239 Department of Family Medicine, University of Washington, Seattle, Washington, USA.

240 Centre for Public Health, University of Iceland, Reykjavik, Iceland.

241 Group Health Research Institute, Group Health, Seattle, Washington, USA.

242 Institute for Translational Genomics and Population Sciences, Los Angeles BioMedical Research Institute at Harbor-UCLA Medical Center, Torrance, California, USA.

243 Centro de Biología Molecular Severo Ochoa (CSIC-UAM), Madrid, Spain.

244 Molecular Genetics Laboratory-Hospital, University of Central Asturias, Oviedo, Spain.

245 Human Genome Sequencing Center, Baylor College of Medicine, Houston, Texas, USA.

246 Department of Internal Medicine, Erasmus University Medical Center, Rotterdam, the Netherlands.

247 Netherlands Consortium on Health Aging and National Genomics Initiative, Leiden, the Netherlands.

248 Arizona Alzheimer's Consortium, Phoenix, Arizona, USA.

249 Banner Alzheimer's Institute, Phoenix, Arizona, USA.

250 Department of Psychiatry, University of Arizona, Phoenix, Arizona, USA.

251 Department of Clinical Sciences, University of Texas Southwestern Medical Center, Dallas, Texas, USA.

252 Departments of Psychiatry, Medicine, and Family and Community Medicine and South Texas Veterans Health Administration Geriatric Research Education and Clinical Center (GRECC), University of Texas Health Science Center at San Antonio, San Antonio, Texas, USA.

253 Institute for Stroke and Dementia Research, Klinikum der Universität München, Munich, Germany.

254 German Center for Neurodegenerative Diseases (DZNE), Munich, Germany.

255 Cambridge Institute for Medical Research, University of Cambridge, Cambridge, UK.

256 Department of Experimental and Clinical Medicine, Neurological Institute, University of Pisa, Pisa, Italy.

257 PharmaTherapeutics Clinical Research, Pfizer Worldwide Research and Development, Cambridge, Massachusetts, USA.

258 Institute of Neurology, Catholic University of Sacred Hearth, Rome, Italy.

259 Departments of Neurology, Pharmacology, and Neuroscience, Texas Tech University Health Science Center, Lubbock, Texas, USA.

260 Department of Psychiatry, Charité University Medicine, Berlin, Germany.

261 Institute of Public Health, University of Cambridge, Cambridge, UK.

262 Department of Medical Sciences, Institute of Biomedicine (iBiMED), University of Aveiro, Aveiro, Portugal.

263 Institute of Psychiatry, Psychology and Neuroscience, King's College London, London, UK.

264 Institute of Brain, Behaviour and Mental Health, Clinical and Cognitive Neuroscience Research Group, University of Manchester, Manchester, UK.

265 3rd Department of Neurology, Medical School, Aristotle University of Thessaloniki, Thessaloniki, Greece.

266 Division of Clinical Neurosciences, School of Medicine, University of Southampton, Southampton, UK.

267 Department of Psychiatry, University of Oxford, Oxford, UK.

268 Florida Alzheimer's Disease Research Center, Gainesville, Florida, USA.

269 Center for Translational and Systems Neuroimmunology, Department of Neurology, Columbia University Medical Center, New York, New York, USA.

270 Department of Health Services, University of Washington, Seattle, Washington, USA.

271 Memory Research and Resources Center, CMRR of Montpellier, Department of Neurology, Hospital Gui de Chauliac, Montpellier, France.

272 Department of Neurology, Montpellier University, Montpellier, France.

273 Memory Research and Resources Center, CMRR de Bordeaux, Bordeaux, France.

274 Neurogenetics Laboratory, Division of Neurosciences, Centre for Applied Medical Research, University of Navarra School of Medicine, Pamplona, Spain.

275 Department of Neurology, Complejo Asistencial Universitario de Palencia, Palencia, Spain.

276 Center for Applied Genomics, Children's Hospital of Philadelphia, Philadelphia, Pennsylvania, USA.

277 Departments of Biology and Neuroscience, Brigham Young University, Provo, Utah, USA.

278 Department of Neurology, Erasmus MC University Medical Center, Rotterdam, the Netherlands.

279 Department of Epidemiology and Biostatistics, Case Western Reserve University, Cleveland, Ohio, USA.

280 University of Bordeaux, Neuroepidemiology, UMR 897, Bordeaux, France.
